# Are You Wearing a Mask? Detecting If a Person Wears a Mask Using a Wristband

**DOI:** 10.3390/s22051745

**Published:** 2022-02-23

**Authors:** Constantino Msigwa, Seungwoo Baek, Denis Bernard, Jaeseok Yun

**Affiliations:** 1 Department of Future Convergence Technology, Soonchunhyang University, Asan 31538, Korea; 20217121@sch.ac.kr (C.M.); 20217120@sch.ac.kr (D.B.); 2 Artificial Intelligence Graduate School, Gwangju Institute of Science and Technology, Gwangju 61005, Korea; swbaek97@gm.gist.ac.kr; 3 Department of Internet of Things, Soonchunhyang University, Asan 31538, Korea

**Keywords:** COVID-19, mask wearing, wristband, accelerometer, IR array, machine learning, deep learning

## Abstract

Coronavirus 2019 (COVID-19) has posed a serious threat to the lives and health of the majority of people worldwide. Since the early days of the outbreak, South Korea’s government and citizens have made persistent efforts to provide effective prevention against further spread of the disease. In particular, the participation of individual citizens in complying with the necessary code of conduct to prevent spread of the infection, through measures such as social distancing and mask wearing, is as instrumental as the geographical tracking of the trajectory of the infected. In this paper, we propose an activity recognition method based on a wristband equipped with an IR array and inertial measurement unit (IMU) to detect individual compliance with codes of personal hygiene management, such as mask wearing, which are recommended to prevent the spread of infectious diseases. The results of activity recognition were comparatively analyzed by applying conventional machine learning algorithms and convolutional neural networks (CNNs) to the IMU time series and IR array thermal images collected from 25 subjects. When CNN and 24 × 32 thermal images were used, 97.8% accuracy was achieved (best performance), and when 6 × 8 low-resolution thermal images were used, similar performance with 97.1% accuracy was obtained. In the case of using IMU, the performance of activity recognition was lower than that obtained with the IR array, but an accuracy of 93% was achieved even in the case of applying machine learning algorithms, indicating that it is more suitable for wearable devices with low computational capability.

## 1. Introduction

According to the statistics presented by the World Health Organization (WHO), the devastating impact of the recent coronavirus 2019 (COVID-19) pandemic led to the number of cumulative confirmed cases reaching 288 million worldwide, as of January 2022, of which approximately 5.4 million patients died [1]. With severe damage caused by COVID-19, individual countries have placed an emphasis on the importance of personal hygiene management, launching campaigns to inform the public of the effective methods of personal hygiene management and to promote compliance to the recommended method. In particular, the Korea Disease Control and Prevention Agency (KDCA) has established a code of conduct for thorough management of personal hygiene to prevent the spread of infectious diseases, such as COVID-19, and recommends that the public abide by the specified code of conduct. Examples include washing hands, avoiding touching the face with unwashed hands, covering the mouth and nose with sleeves when coughing, wearing a mask when going out, and avoiding contact with people with fever and respiratory symptoms. These rules impose restrictions on activities involving high risk of infection [2]. Although the current rate of compliance with the code of conduct is higher than that at the time of the Middle East respiratory syndrome coronavirus (MERS-CoV) pandemic, the rate of noncompliance remains non-negligible, indicating the necessity for improving the rate of compliance with the code of conduct [3].

In line with the current trend, with mounting importance on personal hygiene management to prevent infectious diseases such as COVID-19, it will be beneficial if the status of compliance to the actions prescribed by the code of conduct for personal hygiene management is detected and the information is provided in real-time to the users. At present, due to the possibility of fever as one of the symptoms of COVID-19, body temperature measurement in daily life has become one of the key routines. In Korea, for preemptive screening of the infected on entry to all public buildings, a thermometer using infrared thermal sensor array (infrared array, IR array) is placed and used for measurement of people’s body temperature. However, if the IR array, which is currently fixed in a spot for use is incorporated into a wearable device (e.g., a wristband), it will be possible that people can measure their body temperature without spatial or temporal restrictions. In addition, unlike previous studies that detect movements using thermal images of people acquired from an IR array fixed at a certain location, if the wearers can recognize their own body movements using the IR array mounted on the wearable device, the device can be practically used in the field of activity recognition for personal hygiene management.

**Figure 1:**
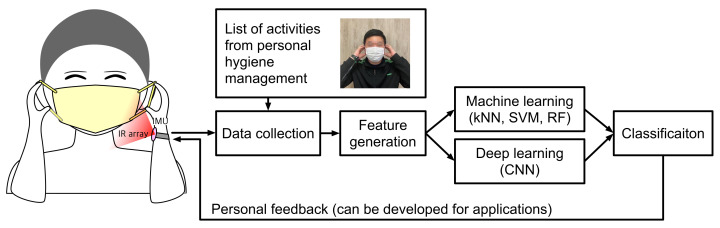
Overall procedure of the proposed method for detecting if the wearer complies with the code of conduct to prevent the spread of infectious diseases.

In this paper, we propose a method for detecting whether the wearer complies with the code of conduct (e.g., mask wearing) to prevent infectious diseases by using a wristband-type wearable device equipped with an IR array and an inertial measurement unit (IMU), as illustrated in Figure 1. A wristband-type wearable device was developed by connecting a six degree-of-freedom (DoF) IMU and an IR array to a commercially available off-the-shelf hardware board to collect data from the wearer’s actions. Based on the actions that are necessary or prohibited for the prevention of infectious diseases as specified and published by KDCA, a list of seven activities that require recognition was derived. Then, data were collected from the IMU and IR array from the actions of 25 subjects. The conventional machine learning algorithms and deep learning methods were applied to the collected data and 10-fold cross-validation was used for a comparative test on the activity recognition performance. From the results of this experiment, with a simple device of wristband equipped with a simple accelerometer or low-resolution IR array alone, effective activity recognition for cost-effective prevention of infectious diseases is possible.

The main contribution of this paper is to propose, for the first time, a recognition method for detecting whether an activity to prevent an infectious disease is performed using a wearable device. In the past, there have been attempts to detect the status of mask-wearing of a subject by taking photographs remotely, using a vision-based sensor such as a camera fixed in a certain spot; however, to the best of our knowledge, in terms of the attempts to detect the compliance with activities related to infectious disease prevention for people with a wearable device, this is the first study that proposes the method. Self-recognition of the status of compliance with activities necessary for infectious disease prevention (e.g., wearing a mask) or, conversely, violation of the code (e.g., touching the mouth with unwashed hands) has significant implications for prevention of the spread of the infectious disease.

With COVID-19 posing a threat to the survival of mankind with devastatingly negative impacts across the global community in economic and social terms, the first basic step for prevention of the spread of the disease is to comply with the code of conduct on personal hygiene management recommended by experts. For the purpose, our method could be worked as a self-monitoring tool that can encourage people to comply with the necessary code of conduct. For example, if the proposed wristband can be implemented with a small vibration motor, it will be able to provide the wristband wearer with an immediate feedback about violation alerts for typical actions. Further, such self-monitoring data could be logged into a wirelessly connected device such as a smartphone and then investigated in further analysis, for example, geographical tracking of the trajectory of the wearer without wearing a mask. In this way, it is expected that the proposed method will serve as a basic means for wise and effective resolution when the global community is faced with yet another unforeseen challenge of infectious disease pandemic in the future.

The remainder of this paper is structured as follows. In Section 2, previous studies on activity recognition using fixed and wearable sensors are reviewed, and the advantages and limitations are presented. Section 3 describes the wearable wristband and data sets collected with the IMU and IR array, and Section 4 describes the learning algorithms used in the activity recognition. Section 5 evaluates the performance of the activity recognition method using the collected data, and Section 6 discusses the limitations and remaining challenges. Finally, Section 7 offers the concluding remarks.

## 2. Related Work

Human activity recognition methods can be divided into two large categories according to whether they use data from sensors installed at fixed locations or not (i.e., wearables).

### 2.1. Fixed Sensors

Fixed sensors are mounted at static locations. Hence, fixed sensor-based activity recognition is confined to limited environments where the sensors are installed [4]; however, they usually take advantage of continuous power supply without needing to replace or recharge batteries.

The most common type of sensors mounted at fixed locations are static cameras. For example, Dhiman and Vishwakarma presented an extensive survey of state-of-the-art techniques for abnormal human activity recognition in images captured from cameras [5]. In particular, depth information obtained from consumer motion sensing cameras such as Kinect is incorporated with RGB data to provide a richer context such as real-time hand tracking. For example, Doan et al. presented a real-time vision-based hand posture recognition system based on a user-guide learning scheme and kernel-based descriptors [6]. Tao et al. presented an American sign language (ASL) alphabet recognition system integrated with multi-view augmentation and inference fusion from depth images captured by Kinect [7].

Thermal images captured from thermal cameras, i.e., IR cameras, have the advantage of eliminating the illumination problems of normal RGB cameras, so a wide range of applications employing thermal cameras have been studied [8], including gender recognition, face recognition, emotion inference, and gait recognition. Wang et al. presented a hybrid gender recognition method by fusing visible and thermal infrared facial images [9]. Seal et al. presented an image fusion algorithm based on the visible and thermal images for face recognition [10]. Wang et al. established a natural visible and infrared facial expression database containing both spontaneous and posed expressions of over 100 subjects [11]. Xue et al. established an infrared thermal gait database by applying the infrared thermal imaging to collect gait video [12]. Recently, Natta et al. presented the rise and regulation of thermal facial recognition technology during COVID-19 pandemic [13].

Aside from abovementioned cameras, millimeter wave (mmwave) radars have been used in gesture sensing and gait recognition. Liu et al. presented a real-time arm gesture recognition system via mmwave sensing for practical smart home usage [14]. Santhalingam et al. proposed an ASL recognition system using a 60 GHz mmwave signals and a scalable and extensible multi-task deep learning model [15]. Meng et al. presented a gait recognition method using mmwave sensing [16].

### 2.2. Wearable Sensors

Wearable sensors would be mainly limited by the lifetime of their power source, i.e., battery; however, for human activity recognition wearable sensors have the advantage that they are wearable and mobile and thus human activities can be monitored in a more first-person perspective [4], almost regardless of their environmental condition. Further, wearable and mobile sensors help to build mobile crowdsensing, which can offer more possibility for data collection during a pandemic [17].

Accelerometers embedded in wearable devices such as smartwatches and smartphones are the most common sensors for activity recognition [18]. For example, Gil-Martin et al. presented a physical activity recognition using a new deep learning architecture and post-processing techniques using hidden Markov models [19]. Stančić et al. presented a hand gesture-based human-robot interface for navigating and controlling a mobile robot in an indoor or outdoor environment [20]. Acceleration signals can also be incorporated with gyroscope outputs to provide an interactive way for interfacing with smartwatches or computers using finger gestures [21], wrist rotation [22], and hand motion such as waving [23].

Acoustic sensors such as microphones are used in wearable devices for activity recognition. Han et al. presented a smartwatch prototype embedded with a microphone array that enables spatial interaction using hand generated acoustic signatures [24]. Zhang et al. presented a sensing technique that can detect fine-grained hand poses by analyzing acoustic resonance features [25]. Cameras (including depth and IR) are also used in the form of wearable sensors for hand gesture recognition [26] and skin input sensing [27]. Recently, physiological signals captured from wearable sensors have been employed, including photoplethysmography (PPG) for finger tap detection [28] and gesture recognition [29] and electromyography (EMG) for forearm interaction [30] and hand gesture classification [31].

### 2.3. Thermal Images from Wearables and Our Motivation

As summarized in the above section, by the advent of low-power computing technologies it has been made possible to employ a variety of sensors in wearable devices to recognize human activities, including inertial sensors, vision sensors, acoustic sensors, and physiological sensors.

Among them, thermal images were used in various applications such as face recognition [32], gender recognition [33], and action recognition [34], but all the applications made use of a set of thermal images captured from static thermographic cameras mounted at fixed locations. Some work employed IR cameras in wearables to classify hand poses [26], but the IR camera images were mainly used to perform accurate background subtraction together with an IR laser line projector and IR LEDs.

During a pandemic such as COVID-19, fever recognition techniques based on a static thermal camera could provide a rapid and noninvasive way to screen for fever in travelers and may be used as an infection control measure [35].

Accordingly, through this article we would like to show the feasibility of thermal images captured from wearable thermal sensors for recognizing human activities, in particular detecting whether the wearer complies with the code of conduct (e.g., mask wearing) to prevent infectious diseases (which, to the best of our knowledge, is not studied previously), with the help of learning algorithms. This is particularly important because the IR thermal sensor is embedded into a wearable device such as wristbands, the wearer will be able to measure his or her body temperature or fever (i.e., as a possible symptom of the infection) in any place at any time, but not limited to some specific areas instrumented with a static thermal camera. Further, as we described in this section, the related work shows the potential of deep learning methods for recognizing human activities using wearable sensor signals. Hence, the question of comparing the performance and effectiveness of learning algorithms, including conventional machine learning and deep learning echoes our motivation in this article.

## 3. Wearable Wristband and Data Collection

### 3.1. Code of Conduct to Avoid COVID-19

**Figure 2:**
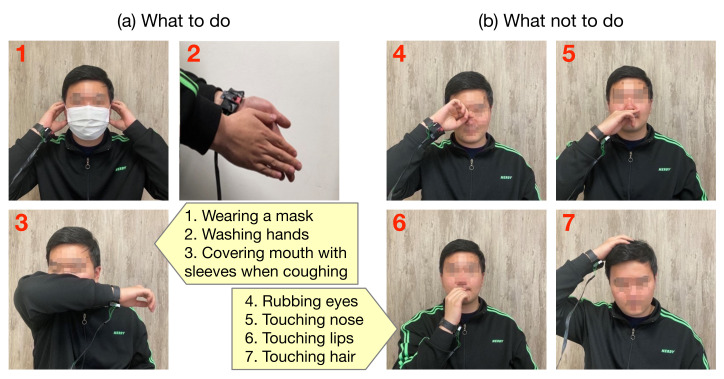
List of activities derived from personal hygiene management code of conduct recommended by KDCA.

The code of conduct on personal hygiene management recommended by the KDCA to prevent the spread of infectious diseases includes the following actions: wearing a mask, washing hands, avoiding touching the face, and covering mouth with sleeves when coughing. From this code of conduct, a list of actions that require recognition in this study was derived, and this is outlined in Figure 2. The recommended codes of conduct for wearing a mask, washing hands, and covering mouth with sleeves when coughing were included in the figure as they are in the code. Further, for the code of not touching the face with unwashed hands, the face area was divided in more detail into eyes, nose, lips, and hair for screening of activities that need recognition when these areas were touched by hands or rubbed (i.e., when the code is violated).

### 3.2. Wearable Data Acquisition System

**Figure 3:**
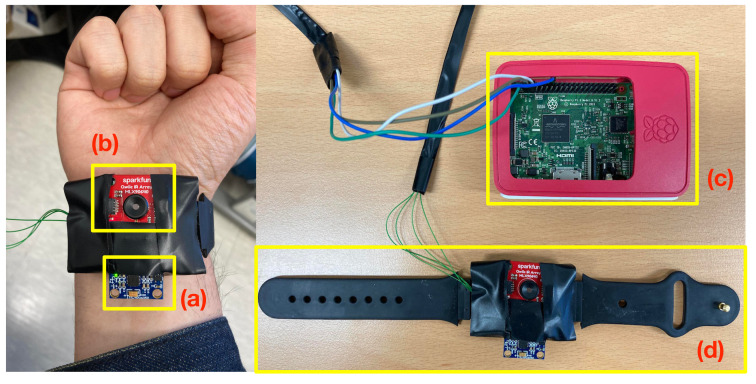
Wearable data acquisition system, (**a**) MPU-6050 (6-DoF IMU), (**b**) MLX90640 (IR array), (**c**) Raspberry Pi 3 B+, and (**d**) wristband.

A wearable data acquisition (DAQ) system that can be worn on the wrist was developed, as shown in Figure 3, to collect data when the wearer performs infectious disease prevention-related actions displayed in Figure 2. The IMU used by most of the existing wearable devices for activity recognition was selected, and an IR array was additionally selected to allow self-examination of the body temperature. For IMU, the MPU-6050 unit capable of measuring 6-axis motion data with a built-in accelerometer and gyroscope was used, and for IR array, MLX90640 capable of acquiring 24 × 32 pixels IR thermal images in a 110${}^{\circ }$ × 70${}^{\circ }$ field of view (FoV) was used. Detailed specification of each sensor is outlined in the following table.

For data collection from the selected sensors, Raspberry Pi 3 Model B+, an open hardware board, was used. As shown in Table 1, each sensor supports the I${}^{2}$C interface as a measured value output; hence, the I${}^{2}$C connector of Raspberry Pi and the sensors were connected. For construction of the DAQ system in the form of a wristband, the inside of a commercial smartwatch was removed (Figure 3d), and the IMU and IR array were placed. In particular, to enable the acquisition of thermal images when the wearer moves the hand wearing the DAQ system, the IR array is placed to face the user on the inner side of the wrist. With this method of sensor placement, FoV of the IR array mounted on the wristband mainly faces the wearer. As a result, when the wearer performs a specific action, the thermal image data including distinguishable features are acquired. For development of an actual practical wearable wristband-type DAQ system, a mobile system with a wireless network module and battery should be used; however, in this study, to focus on the examination on the possibility of activity classification using the IMU data and thermal images collected from the wrist, a DAQ system using cables was developed.

For comparison of activity recognition performance with data collected from the IMU and IR array under the same conditions, the sampling rate of both sensors was set to 8 Hz. Each sensor transmits data measured at a sampling rate of 8 Hz to Raspberry Pi through I${}^{2}$C interface, and finally, in Raspberry Pi, the data received by the DAQ program written in Python are recorded in the micro SD card memory.

### 3.3. Collection of Activity Data

Data were collected from a total of 25 (16 male and 9 female, 23 right-handed and 2 left-handed) participatory subjects using the developed wristband DAQ system. In particular, we tried to collect data from different ages ranging from 22 to 59 years, so the average age of the subjects is 28.9 and the standard deviation is 10.9. Each subject was instructed to perform the seven activities defined in Figure 2 while wearing the wristband DAQ. Each activity was performed 20 times, and measurement data were collected from the IMU and IR array, and a total of 3500 instances (25 subjects × 7 activities × 20 instances) were collected.

### 3.4. Data Sets and Feature Extraction

After a preliminary experiment, we found that subjects spend an average of 4 s wearing a mask. Thus, the time window size for activity recognition was set to be 4 s and zero-padded if necessary.

#### 3.4.1. Inertial Measurement Unit (IMU)

**Figure 4:**
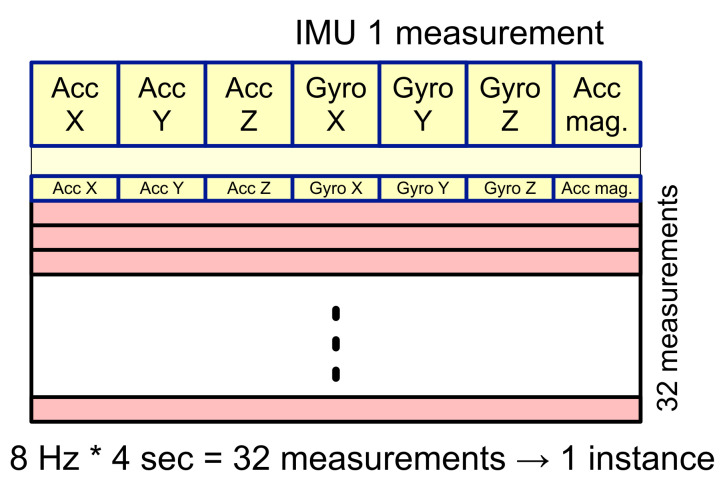
Feature extraction for the inertial measurement unit: three acceleration values at axis x, y, z; three angular rates at axis x, y, z; acceleration vector magnitude.

In the IMU, feature vectors with a total of seven elements, including three acceleration values at x, y, and z axes; three angular rates at x, y, and z axes; acceleration vector magnitude are generated for each measurement (Figure 4). If the IMU performs measurements at a sampling rate of 8 Hz and the duration required to construct one instance for activity recognition is 4 sec, one instance consists of 8 Hz × 4 s = 32 measurements, resulting in a definition of feature vector for a single instance in the form of a 32 × 7 2D array. This 2D array will be reshaped into a 1D array of 224 components, which is subsequently used as an input to machine learning and deep learning models in experiments.

**Figure 5:**
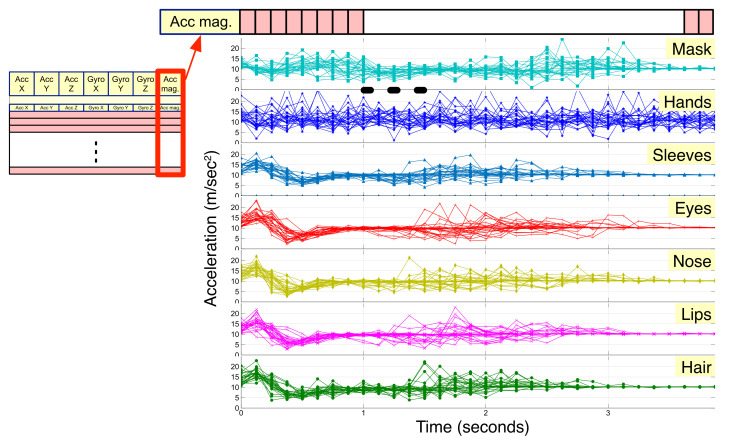
Acceleration vector magnitude signals of 25 subjects for each of the seven activities.

Figure 5 shows the acceleration vector magnitude signals acquired after preprocessing each activity instance collected from the IMU of the wearable DAQ system, while all 25 subjects perform the seven activities selected and listed in Figure 2. It is difficult to distinguish between signals, except for the signal of hand washing. In particular, it can be observed that activities such as eye, nose, and lips rubbing have considerably similar patterns.

#### 3.4.2. IR Array

In the IR array, feature vectors are generated in the form of 2D arrays (i.e., a thermal image) with 24 × 32 pixel values for each measurement. A total of 32 measurements are required if a single instance is composed of the 8 Hz sampling rate and measurement time of 4 sec, as in the case of IMU.

**Figure 6:**
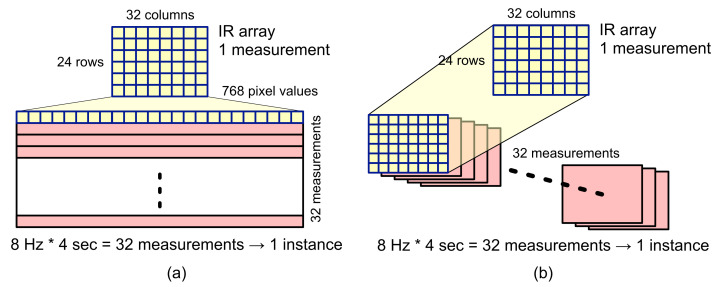
Feature extraction for the IR array (**a**) in the form of 32 1D arrays each containing 768 pixel values and (**b**) in the form of 32 2D arrays each containing 24 × 32 pixel values.

Unlike in the case of the IMU, the IR array shows the measurement result in the form of 2D arrays; therefore, as shown in Figure 6, the feature vectors are constructed in two ways. First, the 24 × 32 pixels of a thermal image output by the IR array for each measurement were flattened to make a 1D array composed of 786 pixel values, and 32 1D arrays were collected to construct one instance (see Figure 6a). As a result, the feature vector for an instance is created in the form of a 32 × 768 2D array. This 2D array will be reshaped into a 1D array and used as an input to machine learning and deep learning experiments.

Second, one instance was constructed by overlapping 32 thermal images obtained for 4 s such as a color channel (or a sequence of gray images) (see Figure 6b). In this method, the feature vector for an instance is created in the form of a 3D array composed of thirty-two 24 × 32 2D arrays. This 3D array can also be used as an input to deep learning experiments. In the subsequent deep learning process of the activity recognition experiment, we present a comparison of recognition accuracy performance according to these different feature vector construction methods.

**Figure 7:**
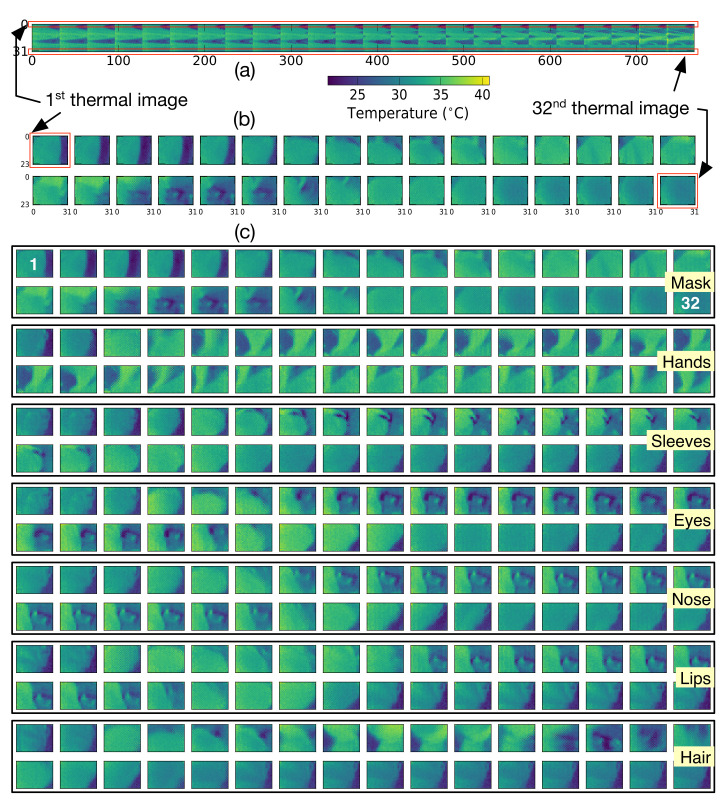
IR array signals collected during mask wearing activity: (**a**) 32 × 768 2D arrays; (**b**) 32 × (24 × 32) 2D arrays; (**c**) 32 2D arrays of one subject for each of the seven activities.

Figure 7a,b show the feature vectors composed using the two methods described in Figure 6 for the pixel values of the instance collected from the IR array, while a subject performs the mask wearing activity. Figure 7a is a 32 × 768 2D array feature vector, where each row corresponding to 32 measurements represents 768 pixels of a flattened thermal image. Figure 7b is a 3D array feature vector, where 32 thermal images containing 24 × 32 pixels representing 32 measurements are constructed as color channels (or a sequence of gray images). Figure 7c shows the 3D array feature vectors captured from one subject for each of the seven activities listed in Figure 2.

## 4. Learning Methods

It is necessary to develop a learning system to perform activity recognition from the collected data. In this paper, conventional machine learning techniques, which have been used for construction of a learning system and verified for the excellent performance in a number of previous studies, and convolutional neural networks (CNNs), which report excellent performance in areas such as image recognition and computer vision in the current research trend, have been used. The performance from the two experiments were compared. Through this comparative analysis, it is possible to determine which learning algorithm is appropriate for activity recognition based on the time series data collected from the IMU and IR arrays. Furthermore, in this paper, the ensemble learning method with soft voting was employed to examine if there is an improvement in the recognition rate when decision fusion is performed using two different models that were trained based on IMU and IR array data.

### 4.1. Machine Learning Algorithms

In classical machine learning techniques, the collected data undergo preprocessing, and features are extracted for learning. In this process, feature engineering, which is the process of identifying the feature that best represents the essence of the data, is crucial; however, in this paper, for comparison with deep learning techniques such as CNN, in which the learning is performed by independently extracting feature vectors from the raw data, without additional process of feature extraction, feature vectors constructed of raw data as described in Section 3 were used.

The selected machine learning algorithms used in this paper are as follows: instance-based learning (i.e., k-nearest neighbor (kNN) algorithm), support vector machine (SVM), random forest, and extremely randomized tree (extra tree). Instead of performing generalization from existing samples, instance-based learning algorithms compare a new input with the known samples and find the closest sample or its class. SVM with linear kernel was chosen, which generates a hyperplane to have the largest margin from the closest sample from each class. Among the families of decision tree, we first chose random forest that generates an ensemble of decision trees trained via randomly sampled data with randomly selected subset of features. A variant of random forest, extra tree was also chosen, where random thresholds are used for each feature rather than performing the best splitting.

### 4.2. Convolutional Neural Network (CNN)

As described above, the most distinct advantage of CNN is that it can independently identify and learn features necessary for performing tasks (e.g., image classification) on large data sets. This is a large advantage compared to previous machine learning systems that required a high level of engineering skill and domain expertise for learning. In particular, in the modern society, where artificial intelligence (AI) is adopted for numerous types of work in everyday lives, CNN enables machines to perform self-learning while minimizing human interpretation.

As the IR array collects an array of thermal values (i.e., 2D thermal images), CNN, which is well-known for good performance in image recognition, was used among a variety of deep learning algorithms. CNN showed a high level of performance in the classification task of 1D time series data, such as signals or sequences as well as 2D array data in previous studies [36]; therefore, good classification performance can be expected when the multimodal data measured using the IR array and IMU in this paper are applied to CNN.

**Figure 8:**
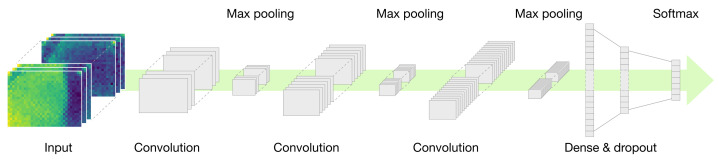
CNN architecture for activity recognition by IR array signals in 32 * (24 × 32) 2D arrays.

Figure 8 shows the CNN architecture used in the activity recognition experiment. Depending on the dimension of the input array, the kernel dimension of the convolutional layer should be set differently. As the IMU data array (i.e., Figure 4) and the flattened IR array (i.e., Figure 6a) have 2D array inputs, 2D convolutional kernels are used in this case. On the other hand, given that the IR array data, composed of successive thermal images, have 3D array inputs (i.e., Figure 6b), 3D convolutional kernels are used. Figure 8 shows the CNN architecture using the 3D convolutional kernel.

The overall CNN architecture in this experiment is as straightforward as standard CNNs in image classification tasks. When the signals measured from the sensor are provided as input to the CNN, they pass through several stages composed of convolutional and max pooling layers, and they are then provided as input to fully connected (FC) layers. In FC layers, dropout regularization is applied to avoid overfitting, and given that the final output is a classification task, the softmax function is used to present the output of the probability of being classified into each activity class. Thereafter, in the ensemble learning experiment, soft voting is performed by using the probability values.

We can vary the convolutional kernel (i.e., filter) sizes or the first few stages of the CNN architecture to obtain the best classification performance. In the subsequent process of the activity recognition experiment, we present a comparison of recognition accuracy performance according to different convolutional kernel sizes in case of IMU signal-based CNN models and different compositions of convolutional and pooling layers in case of IR array signal-based CNN models.

## 5. Experiments and Results

Experiments for activity recognition for personal hygiene management were performed using the collected data set. After performing 10-fold cross-validation with the entire data set, performance metrics such as accuracy, precision, recall, and F1-score were used to compare the performance between selected learning models. In the experiments using machine learning algorithms, Scikit-learn library was used, and in the experiments with CNN, TensorFlow, and Keras libraries were used.

### 5.1. Results with IMU Signals

We performed activity recognition experiment using the machine learning algorithms introduced in Section 4.1 with IMU signals. Table 2 summarizes the parameters that were set for the machine learning models created with the Scikit-learn library, and Table 3 shows the results.

The left four columns represent the experimental results without gyroscope signals (i.e., with the acceleration signals of the IMU alone). If good recognition performance is obtained with signals measured with a 3-axis accelerometer alone without a gyroscope, the economic cost for consumer products can be reduced. As shown in the table, the learning algorithm of extra tree shows the best performance (i.e., accuracy = 91.1%), and the other algorithms (i.e., k-nearest neighbor algorithm, SVM, random forest) show recognition accuracy less than 90%. Other performance metrics such as precision, recall, and F1-score also show results with similar trends in accuracy.

The rightmost column in Table 3 shows the results of the activity recognition experiment obtained using both acceleration signals and gyroscope signals of the IMU. Only extra tree among the chosen machine learning algorithms, which showed the best performance in the experiment with acceleration alone, is applied in this experiment. As indicated in the table, when gyroscope signals are additionally used, the accuracy shows only a small performance improvement (i.e., 1.8% increase) compared to the recognition performance without gyroscope signals. Considering that other performance metrics also show results similar to accuracy, and there is no significant performance improvement when gyroscope signals are added, the use of an inexpensive 3-axis accelerometer is sufficient for activity recognition for personal hygiene management, which is the aim of this paper (i.e., above 91% accuracy).

Next, we performed CNN-based activity recognition experiments with IMU signals. Table 4 summarizes the parameters that were set for the CNN models created with the TensorFlow library.

**Figure 9:**
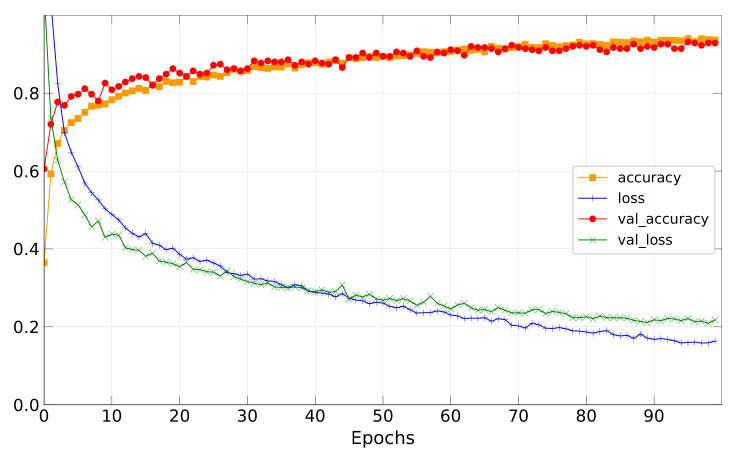
Learning curves: the mean training accuracy and loss, and the mean validation accuracy and loss.

The collected data set has a small number of instances, so we first split it into 90% train and 10% validation set and then applied the CNN model with the parameters in Table 4. Finally, we obtained the learning curves as shown in Figure 9. It should be noted that the validation accuracy curve are steadily increased as the number of epochs increases.

Table 5 compares the two sets of activity recognition results using CNNs according to different kernel sizes, assuming the two cases of using and not using gyroscope signals. Regardless of using gyroscope signals, the larger kernel size is, the better recognition result is. In particular, the CNN with the largest kernel size (i.e., 4 × 4) shows the best performance. When compared with the result in Table 3, CNN shows superior performance to extra tree regardless of whether gyroscope signals are included. Interestingly, when only the acceleration signals of the IMU are used in combination with the CNN, the best activity recognition performance was obtained (i.e., accuracy = 93.5%). On the other hand, the addition of gyroscope signals shows a slight decrease in the performance (i.e., 1.1% decrease); therefore, sufficient performance in the activity recognition for personal hygiene management can be expected using a 3-axis accelerometer alone when CNN is used.

### 5.2. Results with IR Array Signals

The following shows the results of an activity recognition experiment using IR array signals. First, to perform activity recognition with machine learning algorithms, input feature vectors of data set instances were generated in the form of 32 by 768 2D arrays shown in Figure 6a.

Table 6 shows the results of activity recognition experiment using the machine learning algorithms introduced in Section 4.1 with IR array signals. The parameters summarized in Table 2 were also used for the machine learning models with IR array signals. As shown in the table, extra tree shows the best performance (i.e., accuracy = 95.1%), and the rest of the algorithms except for the random forest (i.e., k-nearest neighbor algorithm, SVM) show the performance around 90%. Similar to the experimental results performed using IMU signals, other performance metrics also show trends almost similar to that of accuracy.

The following compares the performance of the results obtained using CNNs with IR array signals according to different number of pairs of convolutional and pooling layers in the first few stages in Figure 8. Additionally, we compare the activity recognition results when generating feature vectors for IR array as in the form of 2D and 3D arrays, as shown in Figure 6. For the experiments, the parameters summarized in Table 4 were also used for the CNN models except epochs = 150.

Table 7 compares the experimental results of CNN with 2D input arrays and CNN with 3D input arrays in terms of accuracy. Regardless of the form of input feature arrays, the more convolutional and pooling layers are, the better the recognition result is. In particular, the CNN with four consecutive pairs of convolutional-pooling layers (i.e., cpcpcpcp) shows the best performance in both two cases.

When input feature vectors are in the form of 3D arrays (i.e., consisting of thirty-two 24 × 32 2D thermal images), CNN showed the better performance (i.e., accuracy = 97.8%) than the result with the input vectors in the form of 2D arrays. Further, this corresponds to an increase in accuracy of 2.7% compared to the extra tree, which showed the best performance among machine learning algorithms shown in Table 6.

The price of IR arrays varies depending on the resolution (i.e., the number of pixels). The higher the number of supporting pixels, the higher the price; therefore, in this study, we examined the difference in the activity recognition result when using an IR array with a lower resolution than the 24 × 32 pixels used in the data set collection process. For this experiment, data sets composed of 12 × 16 thermal images were created by intentionally dropping rows and columns with even-numbered indexes from thermal images composed of 24 × 32 pixels, and the same method was applied once again to create 6 × 8 thermal image data sets.

Table 8 shows the experimental results of activity recognition by applying the extra tree and CNN to the data sets with the three different resolutions generated as above. In both the extra tree and CNN, the recognition rate decreases slightly as the resolution of the IR array decreases (i.e., 0.6% for the extra tree and 0.7% for the CNN when comparing the results between 24 × 32 and 6 × 8 pixels); however, there is little change in the activity recognition performance difference. Consequently, the result indicates that the inexpensive IR arrays with outputs of 6 × 8 pixels thermal images show sufficiently acceptable performance when applied to activity recognition for personal hygiene management.

We performed 10-fold cross validation and found the CNN 3D model with IR array signals outperforms others in terms of accuracy. To provide the validation of this finding, we performed the statistical test for the cross validation results from different algorithms, including cases: C1 (extra tree with acceleration and gyroscope), C2 (CNN with acceleration), C3 (extra tree with IR array), C4 (CNN 2D with IR array), and C5 (CNN 3D with IR array). The number of tests for each algorithm is 10, so Student’s *t*-test is adopted to see that the higher average of accuracy of the CNN 3D model with IR array signal is statistically significant with alpha of 0.05. As summarized in Table 9, all comparisons centering on case 5 exhibit the statistical significance because *p*-values of all tests are much lower than alpha.

Table 10 shows the confusion matrix of 10-fold cross-validation of the CNN with 24 × 32 pixels thermal images model, which showed the best activity recognition performance in Table 8. As shown in the table, most of the classification errors occurred between activities that can be judged to be similar (i.e., rubbing eyes, touching nose, touching lips); however, given that all of these activities can be classified as an activity category that violates the code of personal hygiene management when not wearing a mask, the result indicates that the proposed method can be directly used in cases of personal hygiene management without additional effort to increase the recognition rate (e.g., in case of nose touching, lip touching, or eye rubbing, the wearer is immediately notified of the violation of code).

### 5.3. Results with Multimodal Data (IMU and IR Array)

Finally, ensemble learning was performed to examine whether there is a performance improvement when performing activity recognition using the combination of IMU and IR array. The ensemble learning model was constructed in which the activity is predicted using two extra tree models that showed the best performance in the previous activity recognition experiments performed with IMU (i.e., only acceleration) and IR array, adding the final prediction probability of the respective model and selecting the activity category with the maximum result value to conduct the experiment. The CNN-based ensemble was also constructed as in the extra tree ensemble.

Table 11 shows the accuracy results of activity recognition experiment of the extra tree-based ensemble and CNN-based ensemble, according to the number of pixels in the IR array. As can be observed in the table, the extra tree-based ensemble shows a 1.5% increase in accuracy compared to the case of using only the IR array signals; however, the CNN-based ensemble shows a quite small increase of 0.3% in accuracy. Moreover, similar to the results presented in Table 8, the resolution of the IR array does not significantly affect the recognition accuracy in the ensemble experiment.

## 6. Discussion and Remaining Challenges

When the acceleration and IR array signals collected from the wristband were applied to the CNN models, superior performance was achieved compared to the conventional machine learning algorithms; however, as wristbands are wearable devices with limited battery capacity and computational capabilities, an “on-cloud recognition” method that performs activity recognition by sending feature values collected within a short temporal window to a server or cloud system with learning capabilities through a network can be considered. In the case when the Internet access is not always possible, and “on-device recognition” that performs activity recognition in the wristband is considered, machine learning algorithms that can be implemented with a low-spec system may be more practically feasible than CNN-based methods that require a large amount of computation. In particular, as shown in Table 8, recognition accuracy above 94.5% can be achieved using a 6 × 8 IR array with the lowest resolution and an extremely randomized tree; therefore, for wearable devices such as wristbands, machine learning algorithms can be a more appropriate option than CNN.

By combining the advantage of on-cloud with on-device recognition approach, it would be possible to perform activity recognition of smart wearable devices in a more efficient and flexible way. In general, the most time-consuming parts of any learning algorithm, deep or not, are training models (sometimes including pre-processing and feature extraction). Thus, such parts could be performed on a high-performance cloud system based on the raw data set collected and transmitted from relatively low-power wearable devices via their Internet connectivity such as the 5th generation (5G) network. The wearables have only to download the well-trained model from the cloud and then use it on unseen data for activity recognition. In this way, power-intensive learning algorithms such as CNNs could be also exploited in activity recognition for wearable devices.

For the algorithm of activity recognition, recurrent neural networks (RNNs) that are reported to show good performance in the field of natural language understanding such as speech and text recognition, or long short-term memory (LSTM) networks, which are the variant of RNNs, can be considered. As both the acceleration signals and a sequence of thermal images collected from the wrist are time series data sets, both RNNs and LSTMs can be useful in finding representations that contain essential aspects in differentiating between activities; therefore, if this result is used in combination with CNN models, the recognition performance is expected to improve further.

In this study, we conducted an experiment of activity recognition for data collected only for the “activity of wearing a mask,” but the “activity of taking off the mask” was not included as the scope of the study. The main reason is that, in most cases, users have to use both hands simultaneously to wear a mask, but taking off the mask can be performed with one hand. That is, when the wristband is worn on only one side of the wrist, and the user takes off the mask with the other hand without the wristband, it is very difficult to perform detection and recognition of the activity with the proposed method; therefore, to recognize the activity of taking off the mask, the model can be trained by collecting relevant data while wearing wristbands on both hands (although this is thought to be inefficient), or a separate sensor is placed to the lanyard for face mask, which would assist in detection of the activity of wearing or taking off the mask.

In this study, the activities for which the data were collected and recognized are all activities in which the hand wearing the wristband is raised near the upper body, in particular, close to the head (i.e., personal hygiene practices to be followed to prevent the spread of infectious diseases); however, humans can take various gestures or activities using their hands. Although these various gestures or activities would be difficult to recognize using acceleration signals, they can be recognized with additional thermal images. Hence, the proposed wristband-based recognition method can be applied to recognition of various everyday activities.

## 7. Conclusions

Since the first COVID-19 case was reported in 2019, it has been found that the active participation of citizens in “mask wearing" is crucial to prevent the spread of COVID-19. In this paper, for the detection of the compliance status on the code of conduct on personal hygiene management, a wristband equipped with an IR array and IMU was proposed. A list of seven activities, including mask wearing, was derived among the individual code of conducts for preventing infectious diseases recommended by KDCA. IMU and IR array data were collected from 25 subjects wearing the developed wristband. Among the learning models to perform activity recognition with the collected data set, CNN models outperformed the machine learning algorithms, including extremely randomized trees. In particular, when a 3D array composed of 32 thermal images with 24 × 32 pixel values collected from the IR array was applied to activity recognition with CNN models, the result showed the best accuracy (97.8%), and similar performance (97.1%) was achieved even when the thermal image resolution was reduced to 6 × 8 pixels. The result of the extremely randomized tree model using acceleration signals showed a sufficiently high accuracy of 93%, indicating that this method is suitable for activity recognition in wearable devices with low computational power.

## Figures and Tables

**Table 1:** Specifications for the 6 DoF inertial measurement unit (MPU-6050) and IR array (MLX90640).

	MPU-6050	MLX90640
	(6 DoF IMU)	(IR Array)
Interface	I${}^{2}$C	I${}^{2}$C
Measurement range	±2–±16 G	−40–300 ${}^{\circ }$C
Update rate	4–1000 Hz	0.5–64 Hz

**Table 2:** Model specifications of the machine learning algorithms.

Model	Specifications
knn ${}^{1}$	n_neighbors = 5, weights = ‘uniform’, metric = ‘minkowski’
svm ${}^{2}$	kernel = ‘poly’, degree = 3, C = 5, gamma = ‘scale’
rf ${}^{3}$	n_estimators = 100, criterion = ‘gini’, min_samples_split = 2, min_samples_leaf = 1
xtree ${}^{4}$	n_estimators = 100, criterion = ‘gini’, min_samples_split = 2, min_samples_leaf = 1

^1^ knn: k-nearest neighbor. ^2^ svm: support vector machine. ^3^ rf: random forest. ^4^ xtree: extra tree.

**Table 3:** Performance comparison of machine learning algorithms for activity recognition with IMU signals (mean±std %).

Model	Without Gyro				With Gyro
	knn ${}^{1}$	svm ${}^{2}$	rf ${}^{3}$	xtree ${}^{4}$	xtree ${}^{4}$
Accuracy	86.3±2.0	86.7±2.7	89.4±2.0	**91.1±1.7**	**92.9±1.8**
Precision	87.0±1.4	86.8±2.7	89.7±1.8	91.4±1.6	93.0±1.6
Recall	86.3±1.6	86.8±2.7	89.5±1.8	91.2±1.6	92.9±1.7
F1-score	86.4±1.6	86.6±2.6	89.3±1.8	91.1±1.6	92.8±1.7

^1^ knn: k-nearest neighbor. ^2^ svm: support vector machine. ^3^ rf: random forest. ^4^ xtree: extra tree.

**Table 4:** Model specifications of the CNN model.

Parameter	Specifications
Activation function	activation = ‘relu’
Loss function	loss = ‘sparse_categorical_crossentropy’
Optimizer	optimizer = ‘nadam’
Epoch	epochs = 100
Learning rate	learning_rate = 0.001

**Table 5:** Performance comparison of CNNs according to kernel sizes for activity recognition with IMU signals (mean±std %).

Model	Without Gyro			With Gyro		
	4 × 4 Kernel	3 × 3 Kernel	2 × 2 Kernel	4 × 4 Kernel	3 × 3 Kernel	2 × 2 Kernel
Accuracy	**93.5±1.5**	93.0±0.7	92.6±1.6	**92.4±1.5**	91.9±1.7	91.3±1.7
Precision	93.6±1.5	93.1±0.7	92.7±1.6	92.4±1.4	92.1±1.3	91.6±1.5
Recall	93.6±1.6	93.1±0.9	92.6±1.7	92.4±1.5	92.1±1.3	91.3±1.7
F1-score	93.5±1.6	92.9±0.9	92.5±1.6	92.3±1.5	91.9±1.4	91.2±1.7

**Table 6:** Performance comparison of machine learning algorithms for activity recognition with IR array signals (mean±std %).

Model	knn ${}^{1}$	svm ${}^{2}$	rf ${}^{3}$	xtree ${}^{4}$
Accuracy	89.9±1.6	90.7±0.8	93.7±0.9	**95.1±1.4**
Precision	90.7±1.3	90.7±0.9	93.8±0.9	95.1±1.3
Recall	89.9±1.7	90.7±1.0	93.8±0.8	95.1±1.3
F1-score	89.7±1.7	90.5±0.9	93.7±0.9	95.0±1.3

^1^ knn: k-nearest neighbor. ^2^ svm: support vector machine. ^3^ rf: random forest. ^4^ xtree: extra tree.

**Table 7:** Performance comparison of CNNs according to pairs of convolutional and pooling layers for activity recognition with IR array signals (mean±std %).

Model	CNN 2D			CNN 3D		
	cpcpcpcp ${}^{1,2}$	cpcpcp ${}^{3}$	cpcpc ${}^{4}$	cpcpcpcp ${}^{1,2}$	cpcpcp ${}^{3}$	cpcpc ${}^{4}$
Accuracy	**95.9±1.2**	95.1±1.3	94.9±1.3	**97.8±0.5**	97.3±1.0	96.5±1.1
Precision	96.0±1.2	95.2±1.1	95.1±1.0	97.8±0.5	97.4±0.9	96.6±1.1
Recall	95.9±1.3	95.2±1.3	94.9±1.1	97.8±0.5	97.3±0.9	96.5±1.1
F1-score	95.9±1.3	95.1±1.2	94.9±1.2	97.8±0.5	97.3±0.9	96.5±1.2

^1^ c: convolutional layer, p: pooling layer. ^2^ cpcpcpcp: conv.+pool.+conv.+pool.+conv.+pool.+conv.+pool. layers. ^3^ cpcpcp: conv.+pool.+conv.+pool.+conv.+pool. layers. ^4^ cpcpc: conv.+pool.+conv.+pool.+conv. layers.

**Table 8:** Performance comparison of extra trees and CNNs for activity recognition with IR array signals according to the number of pixels (mean±std %).

Model	xtree ${}^{1}$			CNN 3D		
	24 × 32 Pixel	12 × 16 Pixel	6 × 8 Pixel	24 × 32 Pixel	12 × 16 Pixel	6 × 8 Pixel
Accuracy	**95.1±1.4**	94.8±1.4	94.5±0.9	**97.8±0.5**	97.8±0.8	97.1±0.9
Precision	95.1±1.3	94.8±1.4	94.6±0.9	97.8±0.5	97.8±0.9	97.1±0.9
Recall	95.1±1.3	94.8±1.3	94.6±0.7	97.8±0.5	97.8±0.8	97.1±0.9
F1-score	95.0±1.3	94.7±1.4	94.5±0.8	97.8±0.5	97.7±0.8	97.1±0.9

^1^ xtree: extra tree.

**Table 9:** Student’s *t*-test result of the proposed learning methods.

Statistics	C5 ${}^{5}$ vs. C1 ${}^{1}$		C5 vs. C2 ${}^{2}$		C5 vs. C3 ${}^{3}$		C5 vs. C4 ${}^{4}$	
Mean (%)	97.77	92.86	97.77	93.51	97.77	95.06	97.77	95.89
Variance	0.25	3.56	0.25	2.61	0.25	2.09	0.25	1.56
t Stat	7.966		7.956		5.614		4.427	
P(T <= t) two-tail	1.22 × 10${}^{-5}$ ***		6.88 × 10${}^{-6}$ ***		1.57 × 10${}^{-4}$ ***		8.24 × 10${}^{-4}$ ***	
t Critical two-tail	2.228		2.201		2.201		2.179	

^1^ C1: extra tree with acceleration and gyroscope. ^2^ C2: CNN with acceleration. ^3^ C3: extra tree with IR array. ^4^ C4: CNN 2D with IR array. ^5^ C5: CNN 3D with IR array. *** *p* < 0.001.

**Table 10:** Confusion matrix for activity recognition of the CNN 3D model and IR array signals (i.e., the best recognition rate, 97.8 % with 24 × 32 pixels).

t/p ${}^{1}$	Mask	Hands	Sleeves	Eyes	Nose	Lips	Hair
mask	487	4	2	0	0	1	6
hands	0	500	0	0	0	0	0
sleeves	1	1	494	0	1	2	1
**eyes**	0	0	1	487	10	2	0
**nose**	0	0	0	13	475	12	0
**lips**	0	1	1	4	13	481	0
hair	0	0	0	0	0	0	500

^1^ t/p: true class/predicted class.

**Table 11:** Performance comparison of the ensembles of extra trees and CNNs for activity recognition with IMU (only acceleration) and IR array signals (mean±std %).

Model	xtree ${}^{1}$ Ensemble			CNN Ensemble		
	24 × 32 Pixel	12 × 16 Pixel	6 × 8 Pixel	24 × 32 Pixel	12 × 16 Pixel	6 × 8 Pixel
Accuracy	**96.6±1.2**	96.4±1.0	96.5±0.8	**98.1±0.6**	97.9±0.5	97.5±0.6

^1^ xtree: extra tree.

## Data Availability

Not applicable.
